# White-Seeded Culinary Poppy (*Papaver somniferum* L.) Se Biofortification: Oil Quality, Fatty Acid Profile, and Seed Yield

**DOI:** 10.3390/plants14010095

**Published:** 2024-12-31

**Authors:** Ivana Varga, Tihomir Moslavac, Ivana Flanjak, Dario Iljkić, Milan Pospišil, Zdenko Lončarić, Manda Antunović

**Affiliations:** 1 Department of Plant Production and Biotechnology, Faculty of Agrobiotechnical Sciences Osijek, Josip Juraj Strossmayer University of Osijek, Vladimira Preloga 1, 31000 Osijek, Croatia; dario.iljkic@fazos.hr (D.I.); manda.antunovic@fazos.hr (M.A.); 2 Department of Food Technologies, Faculty of Food Technology Osijek, Josip Juraj Strossmayer University of Osijek, Franje Kuhača 20, 31000 Osijek, Croatia; tihomir.moslavac@ptfos.hr; 3 Department of Food and Nutrition Research, Faculty of Food Technology Osijek, Josip Juraj Strossmayer University of Osijek, Franje Kuhača 20, 31000 Osijek, Croatia; ivana.flanjak@ptfos.hr; 4 Department of Field Crops, Forage and Grassland, Faculty of Agronomy, University of Zagreb, Svetošimunska cesta 25, 10000 Zagreb, Croatia; mpospisil@agr.hr; 5 Department of Agroecology and Environment Protection, Faculty of Agrobiotechnical Sciences Osijek, Josip Juraj Strossmayer University of Osijek, Vladimira Preloga 1, 31000 Osijek, Croatia; zloncaric@fazos.hr

**Keywords:** mineral content, seed quality, yield components, cold-pressed oil, iodine number, linoleic acid

## Abstract

The culinary poppy (*Papaver somniferum* L.) has been used for centuries in everyday diets, especially for food, but also as a non-food source of health-promoting ingredients. In the present study, a field trial was set with white-seeded poppy varieties collected from farmers in Croatia. The poppies were sown as a winter crop. Selenium biofortification was applied using different selenium sources, such as selenite, SeO_3_^2−^, and selenate SeO_4_^2−^. In the flowering stage, biofortification was carried out as follows: (1) Se-0: control; (2) SeO_3__30: 30 g ha^−1^; (3) SeO_3__60: 60 g ha^−1^; (4) SeO_4__30: 30 g ha^−1^; and (5) SeO_4__60: 60 g ha^−1^. Plants formed an average of four capsules per plant, with an average seed mass per capsule of 3.52 g. The seed yield varied from 0.91 (SeO_3__30) to 1.26 t ha^−1^ (SeO_4__30). The cold-pressed oil was characterized as good-quality since the average water content was 0.38%, insoluble impurities consisted of 0.013%, iodine number value was 135.81 g, I_2_ was 100 g^−1^, and saponification number was 188.51 mg KOH g^−1^, and it was on average 0.93% free fatty acids. Selenium biofortification had a significant (*p* ≤ 0.05) impact on Se accumulation in the seeds. Thus, the selenite form increased Se content in the seeds by about 7% compared to the control, whereas for the (4) SeO4_30 treatment, the increase was about 50%, and for (5) SeO4_60, it was even higher, about 91% compared to the control treatment. The highest content of fatty acids in the cold-pressed oil was determined for linoleic (76.31%), oleic (13.49), and palmitic (7.86%) acids.

## 1. Introduction

Cultivated poppy (*Papaver somniferum* L.) is an ancient crop steeped in history that benefits people. The gene center of the poppy is in East Asian (China, Nepal) and Pre-Asian (Asia Minor, Transcaucasia, Iran, highlands of Turkmenistan) territory.

The poppy has been cultivated as a garden and ornamental crop in Europe since the Middle Ages. It is used in the food industry for the unmistakable taste of its seeds and its great culinary properties. The tissues, mainly of the non-mature poppy, can have narcotic effects due to their white latex juice and produce more than 80 alkaloids (morphine, codeine, thebaine, papaverine, etc.) that have been used for thousands of years to soothe the most severe pain, in palliative care, and to treat sleeping problems [1,2,3]. Due to its medicinal properties in the pharmaceutical industry, the poppy is often a part of the drug trade [4].

Culinary poppies are used for seeds and oil extraction. In Southwest Asia, young poppy plants can be used as salad [4]. The colors of the seeds are most often black, grey, and dark blue, but there are also white-yellowish and brown varieties [2]. The content of morphine in dry poppies (poppies with a max. 15 cm stem) generally ranges from almost zero to about 1%, with some varieties having a maximum content of up to 0.8% of morphine alkaloids [5]. There is a constant threat of misuse by people with substance abuse problems. That is why only safe, non-opium poppies are grown for food use in Europe.

Poppy seeds contain 43–45% oil in dry matter and the oil about 28–52%. Cold-press extraction is used for oil, which is used in salads, etc., even though oil production from poppy seeds is quite expensive. Poppy oil is rich in essential fatty acids and contains a dominant 56–75% linoleic acid (C18:2), 10–20% oleic acid (C18:1), and 10–16% palmitic acid [6,7,8,9]. Dąbrowski et al. [7] stated that the composition of fatty acids in poppy oil is not ideal, from a nutritional point of view, due to the predominance of linoleic acid. The fatty acid content depends mostly on the genotype but also on the environment. Satranský et al. [10] argued that poppy cultivars with white seeds contain more linoleic acids compared to blue-seeded cultivars.

In Croatia, the most common oilseeds for oil extraction are sunflower, soybean, and oilseed rape [11,12,13,14]. The cultivation of poppies in the Republic of Croatia is regulated by legislation (NN 18/2012) [15], which allows for the cultivation of poppies by a legal or physical person who has an obligation to report sown areas to the agricultural inspection within 30 days of the sowing date, at the latest.

Croatia and the central part of Europe have a long tradition of growing culinary poppy (*Papaver somniferum* L.), which is used mostly as a seed in bakery products. The most famous cake from this area uses the grey and dark-seeded poppy, called “makovnjača” in Croatian. Nowadays, the largest producer of the culinary poppy in Europe is the Czech Republic, where about 830 farmers grow poppies (average 2018–2022) on approximately 32,400 ha [16]. In Europe, the poppy is cultivated in France, Spain, Hungary, and Germany. The largest producer in the world is Turkey. In Croatia, recently, there has been a growing interest in poppy cultivation, especially in the eastern plain, so it is estimated that about 200 ha is sown poppy.

Still, many family farms in the Republic of Croatia and Europe have old varieties of poppy, used for generations as an integrated part of their culture and heritage. They are most common as baking product decorations or mixed with milk for cookies [17]. It is very valuable to preserve these cultivars and prevent gene erosion.

Across the world, the number of undernourished inhabitants has continued to decrease, but the pace of progress seems insufficient to achieve international hunger reduction goals. According to the last FAO report [18], by 2030, millions of people will still face hunger, and even millions of children will be affected by malnutrition, especially in low-income countries. There have been several attempts to overcome these problems in support of food availability and access and to improve the cost of production and the affordability of food products. Beyond that, the problem of a micronutrient deficit, which is known as the so-called “hidden hunger”, can increase the risk of several diseases. Special attention is paid to preschool-aged children lacking Fe, Zn, and vitamin A, as well as to women of reproductive age lacking Fe, Zn, and folate [19,20].

Selenium is an essential element for humans, and the intake should be 55–200 μg day^−1^ for adults [21]. The specification of the Se lies in the fact that deficiency and toxic concentrations are very close to each other. In the human body, Se is mostly located in skeletal muscles, kidneys, testes, and liver [22,23]. Insufficient uptake of Se can lead to symptoms such as hair loss, abnormal posture, lack of vitality, growth disorder, anorexia, diarrhea, reduced reproductive performance, and fetal deformities [24].

Navarro-Alarcon and Cabrera-Vique [25] stated that approximately 80% of the Se is sourced in the food products and it depends on the diet. One of the efficient agronomic measurements for the prevalence of those issues is the biofortification of crops, intended to increase the levels of microelements in the foodstuffs and, therefore, in the population. Biofortification strategies were developed to produce Se-enriched foods [26,27,28,29,30]. In the soil system, Se is present in inorganic forms such as selenite (SeO_3_^2−^), selenide (Se^2−^), selenate (SeO_4_^2−^), and selenium element (Se).

There is a fast-growing interest in poppy production in Croatia. Little is known about oil quality and the influence of Se biofortification on the content of minerals and oil quality parameters in domestic, white-seeded poppy variety seeds. Nothing is known about oil quality and Se biofortification’s influence on the domestic white-seeded poppy variety seed minerals and oil quality parameters. Thus, this study aimed to evaluate the selenite (SeO_3_^2−^) and selenate (SeO_4_^2−^) foliar application to the seeded poppy variety. Moreover, this study aimed to evaluate oil quality parameters, fatty acid profile, and seed yield with Se biofortification treatment of white-seeded poppy cultivar in Eastern Croatia.

## 2. Results

### 2.1. Yield Components

In the present study, the biofortification treatment with Se did not significantly influence the number of plants per unit area, plant height, number of capsules, or capsule diameter (Table 1). The mass of seeds per capsule was the highest at the SeO_4__60 treatment, which was significantly different from the other mean values of seed mass per capsule. The Se source significantly (*p* < 0.05) influenced the average seed mass per capsule. Thus, Se from Na_2_SeO_4_ was, on average, 3.89, whereas Na_2_SeO_3_ was lower by 0.88 g per capsule on average. The seed yield varied from 0.91 (SeO_3__30) to 1.26 t ha^−1^ (SeO_4__30). The average seed mass per capsule in this study was 3.52 g (Table 1).

### 2.2. Macro- and Microelement Content in the Seeds

This study determined macro- (Cu, Mg) and microelements (Mn, Fe, Cu, Zn, and Se). The Se biofortification treatment significantly influenced seeds’ Mn, Fe, and Se content (Table 2).

Selenium content in the seeds significantly (*p* ≤ 0.05) increased with an increase in the dose of Se with biofortification. It was determined that with the application of the selenite form (SeO_3_^2−^), the average accumulation of the Se in the seed increased by about 7% compared to the control. The selenate (SeO_4_^2−^) application increased Se content in the seeds even more; thus, with the lower dose of 30 g ha^−1^ ((4) SeO4_30), the increment was about 50%, and with 60 g ha^−1^ ((5) SeO4_60), it was even higher, about 91% compared to the control.

**Figure 1:**
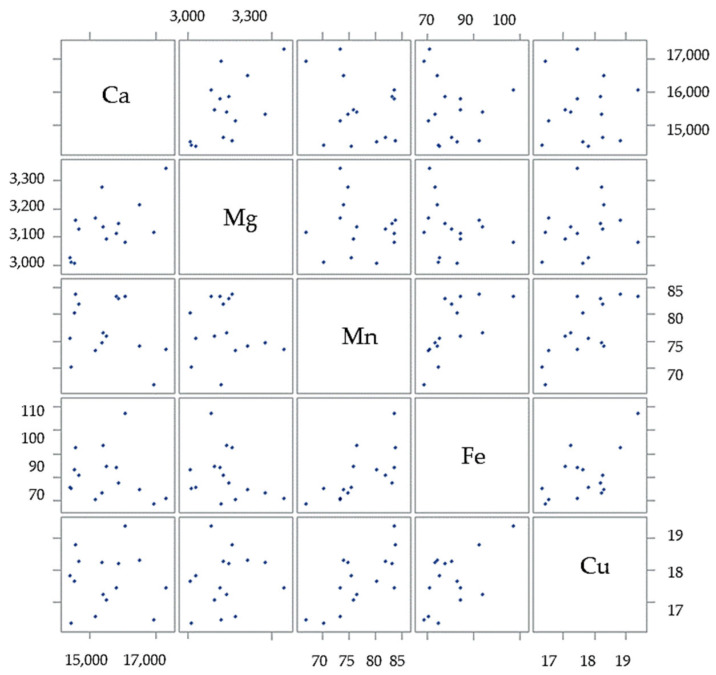
The scatter plot matrix shows the correlation between the micro- and macronutrient status of the white poppy seed.

The correlation analysis showed a significant positive correlation for Ca and Mg (r = 0.609 *), Fe and Mn, Cu and Mn (r = 0.727 **), and Cu and Fe (r = 0.587 *) content in the seed (r = 0.679 **) (Figure 1).

**Figure 2:**
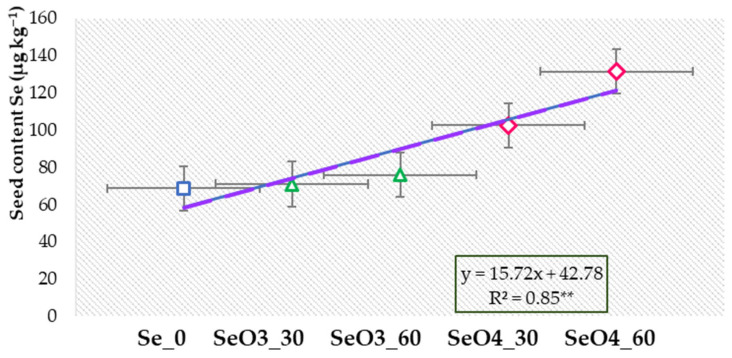
The linear regression of Se source and Se status of the white poppy seed (square represents the control treatment, triangles SeO_3_ and rhombus SeO_4_ treatments). Error bars represent standard error at 5% (*p* < 0.01 is marked as **).

The simple linear regression showed a very significant coefficient of determination (R^2^ = 0.85) for selenium application and Se content in the poppy seeds (Figure 2). Thus, it was found that an increase in Se resulted in an increase in Se in the poppy seed by almost 16 µg kg^−1^.

### 2.3. Amount of Oil and Proteins in the Seeds

Even though there were no significant differences among Se treatments concerning the oil content in the seeds, on the control treatment and with the lower dose of both sources of Se (30 g ha^−1^), the average soil content was higher (50.1%) compared to a higher dose of Se (60 g ha^−1^), where oil content was lower on average by 6.6% (Table 3). The Se treatment did not significantly influence the average protein content, and it was, on average, 22.5%.

### 2.4. Quality Parameters of the Cold-Pressed Oil

**Figure 3:**
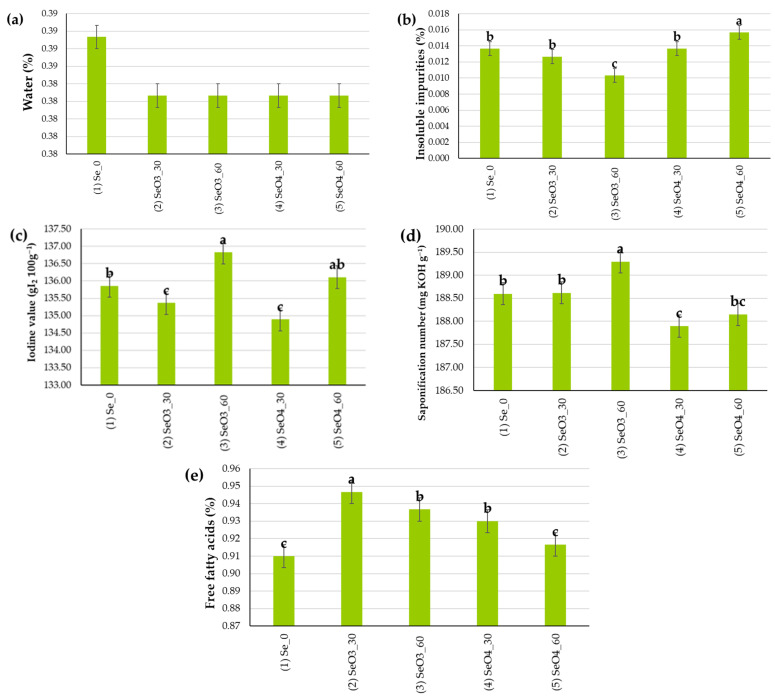
The quality parameters of the cold-pressed poppy oil with regard to Se biofortification treatments: (**a**) water (%), (**b**) insoluble impurities (%), (**c**) iodine value (g I_2_ 100 g^−1^), (**d**) saponification number (mg KOH g^−1^), (**e**) free fatty acids (%). Means with the same letter are not significantly different from each other (*p* < 0.05 ANOVA followed by LSD test). Error bars represent standard error at 5%.

The analysis of the peroxide value (mmol kg^−1^) for all individual samples was 0 mmol O_2_ kg^−1^ because the poppy seeds were fresh and of excellent quality, so after pressing into oil, the value was 0. The Se treatments did not influence the water content of the cold-pressed poppy oil, and it was, on average, 0.38% (Figure 3a).

The Se treatments have a significant influence (*p* < 0.05) on insoluble impurity content (Figure 3b). The average content of insoluble impurities in the present study was 0.013%. The lowest content of insoluble impurities was found under the (3) SeO_3__60 treatment (0.010%), which was 0.06% lower than the (5) SeO_4__60 treatment (0.016%).

The average iodine value in this study was on average 135.81 g I_2_ 100 g^−1^ (Figure 3c) with significant influence (*p* < 0.05). Thus, regardless of the form of selenium, with the highest dose of the Se biofortification applied (SeO_3__60 and SeO_4__60), the highest iodine number value was determined (136.83 and 136.11 g I_2_ 100 g^−1^, respectively), which was on average 0.44% higher compared to the control treatment. On the contrary, the application of 30 g ha^−1^ of Se decreased the iodine number value of the poppy cold-pressed oil by 0.55%, on average, compared to the control treatment.

The average saponification number of this study was 188.51 mg KOH g^−1^ (Figure 3d). The saponification number was the highest under the (3) SeO_3__60 treatment (189.29 mg KOH g^−1^), which was significantly different (*p* < 0.05) in relation to other treatments.

The average content of free fatty acids was 0.93 (Figure 3e). The Se treatments significantly influenced (*p* < 0.05) the free fatty acids in the cold-pressed oil. The highest value was determined with the (2) SeO_3__30 treatment (0.95%), significantly different from other treatments. The lowest content of free fatty acids was determined under the control and (5) SeO_4__60 treatment (0.91 and 0.92%, respectively), with no significant differences among them.

### 2.5. Quality Parameters of the Flour from the Cake

**Figure 4:**
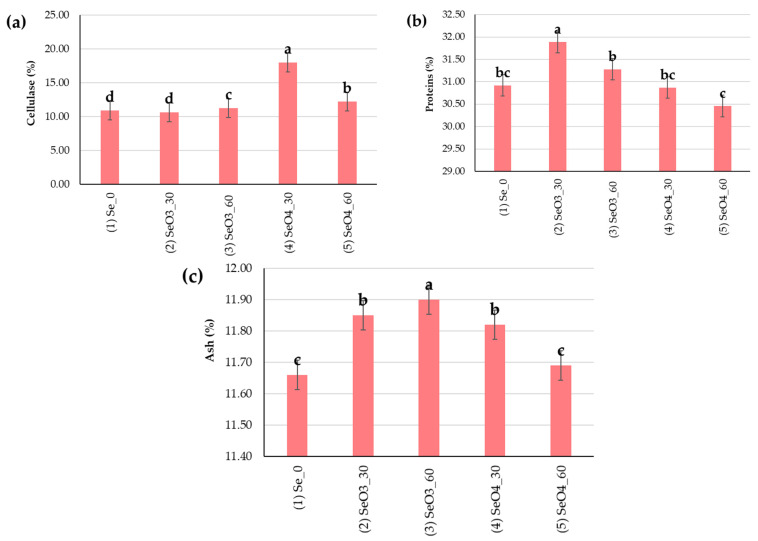
The quality parameters of the flour from the cake with regard to Se biofortification treatments: (**a**) cellulase (%), (**b**) proteins (%), (**c**) ash (%). Means with the same letter are not significantly different from each other (*p* < 0.05 ANOVA followed by LSD test). Error bars represent standard error at 5%.

The average content of cellulase in the poppy flour from cakes was 12.58% (Figure 4a). The highest cellulase content was under (4) SeO_4__30 (17.99%), which was significantly different from the other (*p* < 0.05), and 7.11% higher than the control treatment. The protein content in this study was, on average, 31.08% (Figure 4b). The highest protein content was under (2) SeO_3__30, 0.97% higher than the control treatment (30.92%). The ash content in the poppy cake flour was, on average, 11.78% (Figure 4c). The Se biofortification treatment did not increase ash content in the flour from poppy cake only under the (5) SeO_4__60 treatment.

### 2.6. Fatty Acid Profile 

Based on the fatty acid profile, linoleic acid is the dominant fatty acid in poppy oil (Table 4).

The Se biofortification treatments significantly influenced all the fatty acids, except for palmitoleic acid (C16:1). The highest content of palmitic acid (C16:0) was under (2) SeO_3__30 treatment and the lowest with an increase in SeO_3_ content to 60 g ha^−1^.

Biofortification of Se reduced the content of linoleic fatty acid, whereby the application of form (2) SeO_3__30 reduced linoleic acid by 1.42%, and the application of form (4) SeO_4__30 reduced it by 1.04%. By increasing the biofortification of (3) SeO_3__60 and (5) SeO_4__60, there was an increase in linoleic acid, but the content is still lower compared to the control treatment.

The oleic acid (C18:1n9c+t) was significantly different among each treatment. The increase in the Se biofortification rate significantly increased the oleic acid content with each dose compared with the control treatment (12.90%).

## 3. Discussion

The variability of yields per hectare strongly depends on suitable soil and climatic conditions. The economic yield of poppy is determined by the number of plants per unit area, the number of branches and capsules per plant, and the number of seeds per capsule. According to Kutchova [4], the ideal number of plants at the harvest for organic farming is around 30–40 plants per m^2^ in the sowing, but more often, 18–25 plants per m^−2^ is achieved for spring poppy. The author stated that for the spring poppy, the average is 2–3 capsules per plant. Moreover, the author stated that oversized capsules in poppies provide only a small yield of seeds and that a high yield cannot be achieved with a small number of excessively branched plants because they form more capsules but are smaller in size and elongated shape. In the present study, the average number of capsules per plant was 4, with the capsule diameter around 42 mm. Among six cultivars (Opal, Lazur, Major, Matis, Gornji Bogićevci, and Beli Manastir), Brčić et al. [31] stated that in Croatia (Zagreb), the seed yield varied from 810 kg ha^−1^ of Lazur to 953 kg ha^−1^ of Matis cultivar. The authors found that the average number of capsules per plant was 1.75, with seed mass per capsule (1.59 g).

The oil extracted from the poppy seeds usually contain 40–50% of oil and 10–20% proteins [32]. In the present study, the average oil content in the poppy seed was around 48%, and protein content around 22% (Table 3), per other researchers, defining the oil content as around 40% [7,10,30,33]. Based on the study in Slovakia, Lančaričová et al. [34] stated that for white-seeded poppy genotypes, the oil content can be higher than blue- and grey-seeded genotypes. Based on the analysis of 13 poppy cultivars obtained from the German poppy farmers of different regions, Luhmer et al. [35] reported that oil content from laboratory analysis varied from 33.9% (Mieszko variety) to 48.1% (Zeno Morphex variety). In the Czech Republic (Suchdol area), Satranský et al. [10] found that the oil content of 19 genotypes ranged from 34.56 to 44.76%.

The oil from the poppy seeds predominantly contains linoleic acid, which is a polyunsaturated fatty acid (PUFA). Other fatty acids present in high amounts are saturated and monounsaturated fatty acids (SFAs and MUFAs), respectively, palmitic acid and oleic acid [36]. For the fatty acid content in the poppy oil, Gupcsó et al. [33] in Hungary also found the main fatty acids in the blue- and white-seeded poppy to be linoleic, oleic, and palmitic acids, so the authors stated that the fatty acid profile of the poppy oil is associated more with genotype than with the seed color. Luhmer et al. [35] stated that linoleic acid made up 70.7–75.2% of the fatty acid content in Germany. Erinç et al. [37] argued that the high linoleic acid content in poppy oil makes them unsuitable for oil food products due to their instability and reversion of the flavor associated with auto-oxidation and cold-pressing. Emir et al. [38] utilized descriptive terms, such as roasted, hazelnut, earthy, and waxy, in analyzing the sensory properties of the cold-pressed poppy oil.

In the present study, the basic quality parameter analysis was carried out (insoluble impurities, iodine number, saponification number, peroxide value, and the free fatty acids) on cold-pressed white-seeded poppy oil. The analysis shows that the tested oil is of good quality. The specified parameters are by the Ordinance on Edible Oils and Fats [39,40]. Melo et al. [41] found a total ash content of 10% fresh weight and a total protein content of 26% fresh weight in the poppy cakes. Moreover, the authors stated that poppy cakes could be used as an alternative to animal protein in plant-based diets since they contain all essential amino acids.

In the present study, the Se biofortification significantly influences some parameters of the obtained cold-pressed oil (Figure 3). The iodine value measures the degree of unsaturation in triacylglycerol oils, and it is expressed as the amount of iodine (in grams) that reacts with 100 g of oil. According to Geng et al. [42], high iodine values indicate a higher level of unsaturated fatty acids, which are desirable for nutritional value but may reduce oil stability, while low iodine values reflect a higher proportion of saturated fats, improving stability but potentially lowering nutritional value. Soares and Rocha [43] stated that the iodine value is between 20 and 140 g I₂ 100 g^−1^ for vegetable oils. In the present study, the average iodine value was 135.81 g I_2_ 100 g^−1^, which characterizes it as good-quality oil. Peroxide value quantifies the concentration of peroxides and hydroperoxides formed during the initial stages of lipid oxidation. The low peroxide values indicate good oxidative stability and freshness of the oil, whereas high values suggest oxidative rancidity, negatively affecting flavor, aroma, and healthiness. Maden and Yalçın [44] stated that regarding peroxide value during storage (15–20 °C for 60 days), the increase was greater in white poppy seed fat (15.9%) compared to yellow (11.4%) and gray (9.1%) varieties, which provides important insights into the oxidative stability of these fats. Razay et al. [45] found that due to poppy seed oil’s oxidative stability, the oil has potential in biodiesel production, ensuring longer shelf life and fuel reliability. Free fatty acid content in vegetable oil indicates the hydrolytic degradation of triacylglycerols, whereas high free fatty acids signal a breakdown of triacylglycerols into free fatty acids, which can result in an undesirable taste and reduced quality, and low content is desirable for good-quality oils, as it indicates minimal hydrolysis and spoilage.

Selenium is an essential micronutrient, and the biofortification approach can often overcome the widespread Se deficiency [46]. There are very limited data available on the biofortification of culinary poppy. Skarpa and Richter [47] examined the application of Se to the opium poppy with foliar application of 300 g ha^−1^ Na_2_SeO_3_ at the final stage of the elongation growth and after flowering. The authors stated that the application of Se significantly reduces the seed yield on average, by 11.5% compared to the control, but at the same time, increases the Se content in the seeds from 139 to 757 μg kg^−1^ of seed. The plant species’ specific metabolism determines the organ that accumulates the most Se, the plant tissue’s total amount, and the Se content in the soil’s form [48,49].

Biofortification is often an agronomic measure in cereals such as wheat and rice [50,51,52,53]. For other oilseeds, Se biofortification has been studied more extensively. Garousi et al. [54] applied two forms of Se (Na_2_SeO_3_ and Na_2_SeO_4_) to sunflowers and stated that Se decreased sunflower root growth, but also that translocation of the Se from roots to shoots was higher in sunflowers, as compared to maize. Kurt et al. [55] reported that the Se content of 78 sesame seed samples had a very wide range, i.e., 0–9.32 mg kg^−1^. Except for Se, oilseed biofortification with Zn is common all over the world. For sesame, Eifediyi et al. [56] stated that Zn biofortification (5 to 10 and 15 kg ha^−1^), along with NPK fertilization, increases yield and also Zn content in the seed. The growth stage is considered one of the most important factors of successful biofortification. Dhaliwal et al. [57] reported that the application of borax and urea in combination at critical growth stages (flowering and capsule formation) significantly boosts crop quality, yield, and nutrient uptake of Indian mustard (*Brassica juncea* L.). The authors stated that foliar application has a significant influence (*p* < 0.05) on the moisture, ash, crude fiber, and total soluble solids of the mustard seed. In olive trees (cv. Leccino), D’Amato et al. [58] found that Se fertilization increased the total phenolic content and notably enhanced levels of 3,4-DHPEA-EDA, an oleuropein derivative critical to the antioxidant and health-promoting properties of extra-virgin olive oil.

Selenium is not considered essential to plants but is resilient to various environmental stresses. Even though poppy seeds are mostly consumed directly by humans, the germination of the biofortified Se poppy seeds, especially in drought conditions [59], can be one of the possible ways to evaluate Se biofortification as well. For soybeans, Galić et al. [28] stated that Se-biofortified soybean grain under osmotic stress behaves as a pro-oxidant or antioxidant, depending on the genotype. For sunflowers, Ameen et al. [60] found that Se treatment improved photosynthetic efficiency, plant growth, osmoregulation, and nutrient balance in water-deficit circumstances. Danso et al. [61] stated that under extreme biofortification of lamb’s lettuce with Se, both foliar and soil (50 mg Se/dm^3^ Na_2_SeO_4_) improved plant growth and reduced oxidative stress.

Since the poppy seeds are directly consumed, mostly in bakery products, this research gives insight into Se biofortification, with the special aim of enriching white poppy seeds. Generally, the Se biofortification increased significantly (*p* < 0.05) the Se content in the poppy seed. Cold-pressed poppy oil has good quality parameters and great potential for a plant-based diet, as well as cakes for human consumption as flour or as feedstuff for animals.

## 4. Materials and Methods

### 4.1. Field Experiment Set-Up

The white poppy seed was sown as a winter crop. The experiment was set up in Eastern Croatia at the Experimental station of the Faculty of Agrobiotechnical Sciences Osijek at the “Tenja” location. This poppy variety was stored in the National Bank of Plant Genes, whereas their passport data can be found in the Croatian Plant Genetic Resources Database [62].

Sowing was performed manually on 7 October 2022. During the growing season, crop thinning was performed on three occasions: manual hoeing of crops.

The foliar biofortification with liquid selenium (1 June 2023) was carried out in the flowering phase. There were five different treatments of Se application in the flowering stage (Table 5).

### 4.2. Weather Data

Young poppy plants are resistant to cold and frost during growth. However, their requirements for warmth and durability change as they develop. Temperature, along with sufficient moisture, affects germination speed and emergence time. Until the formation of a rosette of leaves, the resistance of the plants increases. The temperatures at the time of sowing (October) were not critical for emergence, so despite the lack of precipitations (Table 6), plants established satisfied plant populations until December. Generally, the winter period was warm, especially in January, when the temperatures were 3.9 °C higher than the long-term mean (LTM).

### 4.3. Plant Samples

Plants were harvested manually by 2 m^2^ for each repetition on 18 July 2023. There was a total of three repetitions for each Se biofortification treatment. After harvest, a total of 10 plants from each repetition were chosen randomly to determine yield components of the white-seeded culinary poppy variety: no. of plants ha^−1^, plant height (cm), no. of capsules per plant, capsule diameter (mm), the mass of seed per capsule (g), and total seed yield (t ha^−1^).

### 4.4. Macro- and Micronutrient Status in the Poppy Seeds

After harvest, seeds were dried and milled. Digestion of the sample was determined in the microwave (Sineo Microwave Digestion, model Tank 40, Shanghai, China), with the 0.2 g weighing of plant material with ramping stage: 10 min. 150 °C and 25 min. 190 °C. Each sample was combined with 6 mL of HNO3 and 2 mL H2O2 (Ficher Scientific, Hampton, NH, USA) and, after digestion, transferred to centrifuge tubes where they were topped up to 50 mL with ultrapure water. The concentration of elements was manifested on the ICP-MS 7800 Agilent Technologies (Santa Clara, CA, USA) device.

### 4.5. Oil and Proteins in the Seeds

The content of the crude oils in the dry matter of the sample in the seeds was determined using the Soxhlet [64]. Samples were weighed at 3 g with 130 mL petrol ether added into a glass and put for 120 min at Buchii Extraction System B-811 (extraction).

For protein content determination, poppy seeds were dried and milled. The poppy seed samples were weighed to 100 mg in ceramic containers and then placed in the autosampler device C/N analyzer (Scalar Primacs SNC-100-IC, Bayern, Germany). The device dry burns the samples at 1200 °C and determines the N concentration according to the Dumas method. The protein quantification in the poppy seeds was determined by multiplying the value of the N content by 6.25.

### 4.6. Production and Oil Quality Parameters

Cold-pressed white poppy seed oil was produced using a screw press with an electric motor power of 1.5 kW. Crude oil was obtained by pressing poppy seeds clarified by sedimentation and vacuum filtration. As a by-product of pressing, the cake was ground into flour, which is also used in the food industry. Afterward, the International Organization for Standardization determined peroxide value, water content, insoluble impurity content, iodine number value, and saponification number [65,66,67,68]. After pressing the oil, the cellulase, ash, and protein content of the cake’s remains were determined.

The peroxide value (mmol O_2_ kg^−1^) indicates the degree of oxidative spoilage of edible vegetable oils. The result is expressed as mmol of active oxygen originating from the peroxides in 1 kg of oil. The value is calculated according to the equation:

Peroxide value (mmol O_2_ kg^−1^) = (*v*1 − *v*0) 5/*m* (1)

where:

*v*1 = volume of sodium thiosulfate solution;
*v*0 = volume of sodium thiosulfate solution used for blank titration (mL);
*m* = mass of oil sample (g).

The water content of the poppy cold-pressed oil was calculated according to the expression:

(2) $$\mathrm{Water}\;\mathrm{content}\;(\%)=\frac{m1-m2}{m1-m0}\times 100$$

where:

*m*0—mass of the glass container (g);
*m*1—mass of glass container and sample before drying (g);
*m*2—mass of glass container and sample after drying (g).

The share of insoluble impurities is calculated according to the expression:

(3) $$\mathrm{Share}\;\mathrm{of}\;\mathrm{insoluble}\;\mathrm{impurities}\;(\%)=\frac{m2-m1}{m0}\times 100$$

where:

*m*0—sample mass (g);
*m*1—mass of the dried funnel;
*m*2—mass of the funnel with impurities after drying (g).

Determining the iodine number indicates the unsaturation of oil or fat. The iodine number represents the amount of iodine in grams that binds to 100 g of oil or fat. The iodine number is determined according to the formula:

(4) $$\mathrm{Iodine}\;\mathrm{number}\;(g\;100\;g^{-1})=\frac{\left(v0-v1\right)\cdot 0.01269}{c}$$

where:

*v*0 = volume of used 0.1 M sodium thiosulfate solution for blank titration (mL);
*v*1 = volume of used 0.1 M sodium thiosulfate solution for sample titration (mL);
*c* = mass of the tested sample (g).

The saponification number indicates the number of mg of KOH required for the complete saponification of free and ester-bound fatty acids in 1 g of fat. The saponification number is determined according to the formula:

$$\mathrm{Saponification}\;\mathrm{number}\;(\mathrm{mg}\;\mathrm{KOH}\;g\;{\mathrm{oil}}^{-1})=\frac{\left(v0-v1\right)\cdot 2.81}{m}$$

*v*0 = volume of 0.5 M HCl solution used for blank titration (mL);
*v*1 = volume of 0.5 M HCl solution used for sample titration (mL);
*m* = sample mass (g);

1 mL of a 0.5 M HCl solution is equivalent to 28.1 mg of KOH.

### 4.7. Determination of Fatty Acid Profile 

The fatty acid methyl esters (FAMEs) were prepared with cold methanolic potassium hydroxide solution according to the procedure described in Annex X B of the Commission Regulation No 796/2002 [69]. FAMEs were afterward separated on a Shimadzu GC-2010 Plus (Kyoto, Japan) gas chromatograph equipped with a flame ionization detector (FID) and fitted with a SH-FAMEWAXTM (Kyoto, Japan) capillary column (30 m, 0.32 mm i.d., and 0.25 µm thick stationary phase). Nitrogen was used as a carrier gas, flowing at the constant linear velocity of 1.26 mL/min. The split/split less injector was set at 240 °C, the split ratio was 1:100, and the injection volume was 2 µL. The initial column temperature of 120 °C was held for 5 min, then gradually increased to 5 °C/min until the temperature of 220 °C was held for 20 min. The flame ionization detector temperature was 250 °C. Identification of separated FAMEs in samples was achieved based on the comparison of retention times with the retention times of a certified reference standard (Supelco F.A.M.E. Mix, C4-C24, St. Louis, MO, USA) analyzed under the same conditions. The results were expressed as a percentage of identified fatty acids on total fatty acids (%).

### 4.8. Statistical Analysis

All data were subjected to the analysis of variance (ANOVA) as a one-way ANOVA procedure with SAS Enterprise Guide 7.1 [70]. The differences between the means were calculated with the Student *t*-test (LSD test) and presented at *p* < 0.05. The correlation analysis was calculated using Pearson’s coefficient of correlation.

## 5. Conclusions

Biofortification is a process by which the concentration of essential elements in the edible part of harvested products is increased through agronomic intervention. This first study gives comprehensive insights into Se biofortification in Croatian white-seeded poppy, as well as oil quality and fatty acid profile. Generally, the Se biofortification increased Se content in the seeds. Since only limited data are available for poppy biofortification with Se, this research is valuable as the first recorded study of Se biofortification in Croatia for further experiments with white-seeded poppy and other varieties.

## Figures and Tables

**Table 1:** The yield and yield components of opium poppy in relation to Se biofortification.

Treatment	Plant Height (cm)	No. of Capsules Per Plant	Capsule Diameter (mm)	Mass of Seed Per Capsule (g)	Seed Yield (t ha^−1^)
(1) Se_0	139.6	3	43.5	3.82	1.23
(2) SeO_3__30	140.4	3	41.5	3.00	0.91
(3) SeO_3__60	138.9	4	42.1	3.02	1.31
(4) SeO_4__30	143.0	4	41.5	3.48	1.26
(5) SeO_4__60	142.1	4	42.5	4.29	1.02
Mean	140.8	4	41.7	3.52	1.15
LSD (0.05)	ns	ns	ns	0.95	0.35

**Table 2:** Macro- and microelement content in opium poppy seed in relation to Se biofortification.

Treatment	Ca (mg kg^−1^)	Mg (mg kg^−1^)	Mn (mg kg^−1^)	Fe (mg kg^−1^)	Cu (mg kg^−1^)	Zn (mg kg^−1^)	Se (µg kg^−1^)
(1) Se_0	15,283	3082	78.5	92.2	18.1	55.3	68.7
(2) SeO_3__30	16,317	3242	73.6	72.5	17.4	54.4	71.0
(3) SeO_3__60	15,440	3235	82.8	86.7	18.1	54.7	75.9
(4) SeO_4__30	16,317	3130	70.6	72.3	17.0	52.5	102.6
(5) SeO_4__60	14,843	3088	82.8	81.0	17.8	56.8	131.5
Mean	15,491	3135	77.1	80.9	17.7	54.7	89.9
LSD (0.05)	ns	ns	5.8	14.1	ns	ns	14.6

**Table 3:** The oil and protein content of opium poppy seed in relation to Se biofortification.

Treatment	Oil Content (%)	Protein Content (%)
(1) Se_0	49.5	22.6
(2) SeO_3__30	51.1	22.1
(3) SeO_3__60	43.5	22.8
(4) SeO_4__30	49.6	22.5
(5) SeO_4__60	43.5	22.6
Mean	47.5	22.5
LSD (0.05)	ns	ns

**Table 4:** Mean values and standard deviation (SD) of fatty acid composition (%) of white-seeded poppy oil in relation to biofortification treatments.

	Palmitic (C16:0)	Palmitoleic (C16:1)	Stearic (C18:0)	Oleic (C18:1n9c+t)	Linoleic (C18:2n6c)	α-Linolenic (C18:3n3)	Lignoceric (C24:0)
(1) Se_0	7.89 ± 0.08 ^ab^	0.11 ± 0.01	1.63 ± 0.00 ^ab^	12.90 ± 0.05 ^e^	76.84 ± 0.13 ^a^	0.50 ± 0.01 ^a^	0.13 ± 0.01 ^a^
(2) SeO_3__30	7.95 ± 0.04 ^a^	0.11 ± 0.00	1.62 ± 0.01 ^ab^	13.97 ± 0.04 ^a^	75.75 ± 0.07 ^c^	0.48 ± 0.01 ^b^	0.12 ± 0.01 ^a^
(3) SeO_3__60	7.79 ± 0.02 ^b^	0.11 ± 0.00	1.64 ± 0.02 ^a^	13.78 ± 0.03 ^b^	76.16 ± 0.05 ^b^	0.50 ± 0.00 ^a^	0.11 ± 0.01 ^b^
(4) SeO_4__30	7.88 ± 0.04 ^ab^	0.11 ± 0.00	1.62 ± 0.01 ^ab^	13.70 ± 0.02 ^c^	76.04 ± 0.04 ^b^	0.50 ± 0.01 ^a^	0.13 ± 0.00 ^ab^
(5) SeO_4__60	7.85 ± 0.09 ^ab^	0.11 ± 0.00	1.61 ± 0.01 ^b^	13.08 ± 0.03 ^d^	76.75 ± 0.09 ^a^	0.50 ± 0.00 ^a^	0.11 ± 0.01 ^b^
Average	7.86	0.11	1.62	13.49	76.31	0.50	0.12

Means with different letters in the same row are significantly different (LSD test at *p* ≤ 0.05).

**Table 5:** Treatments of Se biofortification.

Treatment	Source of Se	Dose of Se (g ha^−1^)
(1) Se_0	Control	0
(2) SeO_3__30	Na_2_SeO_3_	30
(3) SeO_3__60		60
(4) SeO_4__30	Na_2_SeO_4_	30
(5) SeO_4__60		60

**Table 6:** The average temperatures (°C) and precipitations (mm) at the location Osijek [63].

Month	Temperature (°C) 2022–2023	Temperature (°C) LTM	Precipitations (mm) 2023–2022	Precipitations (mm) LTM
October	13.6	12.0	12.0	59.3
November	7.9	7.1	78.0	51.4
December	4.7	2.4	59.8	48.1
January	4.7	0.8	65.3	44.0
February	3.8	2.5	53.5	46.3
March	8.8	7.0	27.9	42.4
April	10.9	12.8	76.0	47.2
May	17.3	17.5	99.2	81.4
June	21.3	21.8	51.8	75.6
July	24.2	23.4	56.3	58.6

## Data Availability

Data are contained within the article.
